# Genomic Characterization of Clinical *Listeria monocytogenes* Isolates in Beijing, China

**DOI:** 10.3389/fmicb.2021.751003

**Published:** 2021-12-10

**Authors:** Xiaoai Zhang, Yuzhu Liu, Penghang Zhang, Yanlin Niu, Qian Chen, Xiaochen Ma

**Affiliations:** ^1^Institute for Nutrition and Food Hygiene, Beijing Center for Disease Prevention and Control (CDC), Beijing, China; ^2^Beijing Research Centre for Preventive Medicine, Beijing, China

**Keywords:** genomic characterization, *Listeria monocytogenes*, clinical isolates, virulence islands, resistance genes

## Abstract

*Listeria monocytogenes* is a foodborne human pathogen that affects public health worldwide. Whole-genome sequencing (WGS) can classify *L. monocytogenes* isolates and identify virulence islands and resistance genes potentially influencing infectivity. Herein, WGS was used to assess 151 *L. monocytogenes* isolates from 120 cases of clinical infection in Beijing, China, between 2014 and 2018. Most isolates were either serogroup 1/2a,3a or serogroup 1/2b,3b,7, with 25 multilocus sequence typing (MLST) types (STs) represented, of which ST8, ST87, and ST5 were the most common. Core-genome MLST (cgMLST) grouped the 151 isolates into 116 cgMLST types. The discriminatory power of cgMLST was greater than other subtypes, revealing that isolates from the same patient were highly related (only differing at one allele). Eighty-six isolates formed 30 complexes with ≤ 7 cgMLST alleles between neighboring isolates, suggesting possible outbreaks. Compared with isolates in the United States, ST8, ST121, ST619, ST87, and ST155 isolates were grouped into unified clades. All 151 isolates were positive for common virulence-associated loci, and 26 lineage I isolates harbored the pathogenicity island 3 (LIPI-3) locus, while 42 lineage I isolates harbored the complete LIPI-4 locus. Eleven ST619 isolates had both LIPI-3 and LIPI-4. Among the 151 isolates, 13 were resistant to at least one antibiotic, and no multidrug-resistant isolates were identified. Resistance phenotypes correlated with genotypes, apart from two meropenem resistance isolates. The findings provided insight into the nature of *L. monocytogenes* strains currently causing clinical disease in Beijing, and WGS analysis indicated possible outbreaks.

## Introduction

*Listeria monocytogenes* ubiquitous in the environment and is a major foodborne pathogen affecting public health (de Noordhout et al., 2014). It causes listeriosis, a severe infection characterized by sepsis, meningitis, pregnancy loss, and can even be fatal to immuno-compromised or older patients. Listeriosis accounts for a disproportionate share of the foodborne disease burden, with high hospitalization and fatality rates (Scallan et al., 2011; de Noordhout et al., 2014), ranging from 15 to 30%, the highest among all foodborne infectious diseases (Barton Behravesh et al., 2011; Scallan et al., 2011; Hernandez-Milian and Payeras-Cifre, 2014). As a special pilot project of the National Foodborne Disease Surveillance Plan, human listeriosis surveillance has been implemented in 2013, and the fatality rate of listeriosis was 26.1% in China during 2013−2017 (Li et al., 2019).

*L. monocytogenes* causes sporadic cases or protracted outbreaks, and even multi-country outbreaks, and often the specific source may not be known (Halbedel et al., 2018). A subtyping method with high resolution, reproducibility, and exchangeability is required for international surveillance and investigation. Different methods are used to subtype *L. monocytogenes*. Pulsed-field gel electrophoresis (PFGE), the typing golden standard, has been used internationally, but lacks comparability between different networks and sufficient discriminatory power, and does not reflect the evolutionary relationships among strains (Gerner-Smidt et al., 2006). The other standardized genotyping method, multilocus sequence typing (MLST) based on seven genes, is an internationally comparable subtyping method, but lacks the discriminatory power required for epidemiological surveillance (Ragon et al., 2008; Chenal-Francisque et al., 2011; Haase et al., 2014). Whole-genome sequencing (WGS), a powerful epidemiological typing tool, can differentiate isolates that are indistinguishable by other typing methods (Harris et al., 2010; Mutreja et al., 2011; Maury et al., 2016; Moura et al., 2016). Thus, WGS has been widely applied for investigating outbreaks and contamination of food production plants (Schmid et al., 2014; Stasiewicz et al., 2015; Bergholz et al., 2016; Kwong et al., 2016). WGS can also help to predict pathogenic loci in virulent or hypervirulent strains. Furthermore, WGS can reveal population structure and infer evolutionary relationships among strains from a wide range of geographic, temporal and epidemiological origins (Moura et al., 2016).

*L. monocytogenes* encodes internalins and genomic islands (Listeria pathogenicity islands, LIPIs), that play important roles in pathogen virulence. Internalins help *L. monocytogenes* invade host cells (Gaillard et al., 1991). Some *L. monocytogenes* strains isolated from environment and food sources produce a truncated form of the InlA protein, and virulence is reduced (Jacquet et al., 2004; Nightingale et al., 2008). LIPI-1, the main pathogenicity island, is well conserved across strains independent of lineage (Gouin et al., 1994). LIPI-3, encoding a second haemolysin known as listeriolysin S (LLS), is strongly associated with lineage I strains (Cotter et al., 2008). LIPI-4, which includes a cellobiose family phosphotransferase system, is strongly associated with certain lineage I strains that are associated with invasion of the central nervous system (Maury et al., 2016).

In this study, we used WGS to assess the genomic diversity of *L. monocytogenes* in Beijing, China, to compare methods (PFGE, MLST, cgMLST, and wgSNP) for determining relatedness of the isolates. We also aim to characterize the distribution of virulence determinants of *L. monocytogenes*, to detect the absence/presence of antimicrobial resistance-encoding genes and their relationship with antimicrobial resistance profiles.

## Materials and Methods

### Bacterial Isolates

Human listeriosis surveillance in Beijing, as a special pilot project of the National Foodborne Disease Surveillance Plan has been implemented since 2013. In this surveillance, all the suspected clinical cases of listeriosis were included in the survey. Samples were collected and used to isolate *L. monocytogenes.* We defined invasive listeriosis as isolation of *L. monocytogenes* strains from a normally sterile site or from products of conception (Li et al., 2018). A total of 129 human patients who had a severe illness with serious suspicion of *L. monocytogenes* infection were supervised between 2014 and 2018. Among the 129 human patients, 151 isolates were isolated from 120 human patients, while the isolates in other 9 cases were lost. All *L. monocytogenes* isolates identified by clinical microbiology laboratories were sent to the Beijing CDC lab. All isolates were firstly identified using a VITEK 2-compact System (bioMérieux, Lyons, France) or matrix-assisted laser desorption/ionization time of flight (MALDI-TOF) mass spectrometry (Bruker, Leipzig, Germany).

Furthermore, the genome sequences, which were downloaded from Genbank, of 368 human *L. monocytogenes* strains from the United States were used for comparing with that of our isolates (Moura et al., 2016). The 368 strains were isolated between 2009 and 2014. The information of these strains were shown in Supplementary Table 1.

### Serotyping, Multilocus Sequence Typing and Pulsed-Field Gel Electrophoresis Analyses

*L. monocytogenes* isolates were serotyped by multiplex PCR assay (Doumith et al., 2004). MLST analysis was performed by sequencing seven housekeeping genes. Alleles and sequences types (STs) were determined by comparison with allelic profiles for *L. monocytogenes* in the MLST database.^1^ For PFGE analysis, the *Asc*I restriction enzyme was used according to the PulseNet International protocol (Almeida et al., 2017).

### Antimicrobial Susceptibility Testing

Antimicrobial susceptibility testing of *L. monocytogenes* isolates was performed using the broth dilution method. We measured the minimum inhibitory concentrations (MICs) of ampicillin (AMP), penicillin (PEN), tetracycline (TET), meropenem (MRP), trimethoprim-sulfamethoxazole (SXT), erythromycin (ERY), vancomycin (VAN), and ciprofloxacin (CIP) (Xingbai, Shanghai, China). The MICs of AMP, PEN, SXT, and MRP were interpreted using the Clinical and Laboratory Standard Institute (CLSI) International Guidelines, and the MIC of ERY was interpreted according to European Committee on Antimicrobial Susceptibility Testing (EUCAST) International Guidelines. Resistance criteria have not been reported for TET, VAN or CIP for *L. monocytogenes*, hence, the MICs of these three antimicrobials were interpreted using those recommended for *Staphylococcus* spp. ATCC29213, which was used as the reference strain.

### Whole-Genome Sequencing

Isolates of *L. monocytogenes* were routinely grown in brain heart infusion broth overnight at 37°C. DNA was extracted using a DNeasy UltraClean Microbial Kit (Qiagen, Germany). Sequencing was performed using an Illumina Novaseq apparatus (Illumina Inc., San Diego, CA, United States) and constructing two paired-end (PE) libraries with average insertion lengths of 350 and 2,000 bp, respectively. Raw data were processed in four steps, including removing reads with 5 bp of ambiguous bases, removing reads with 20 bp of low quality (≤ Q20) bases, removing adapter contamination, and removing duplicated reads. Finally, 100 × libraries were obtained with clean PE read data.

### Phylogenetic Analysis

The WGS raw data were imported into BioNumerics software (version 7.6 Applied Maths, Kortrijk, Belgium), then uploaded to the National Molecular Tracing Network for Foodborne Diseases Surveillance (TraNet) calculation engine at aliyun for *de novo* assembly, core genome MLST (cgMLST), and whole-genome single-nucleotide polymorphism (wgSNP) analyses using default settings (Li et al., 2021). The cgMLST scheme included 1748 loci for *L. monocytogenes* in BioNumerics. The wgSNP analysis was further carried out on the 86 isolates with cgMLST ≤ 7 different alleles. F2365, EGDe and ICDC_LM188 were chosen as reference genomes for 4b,4d,4e, 1/2a,3a, and 1/2b,3b,7 serogroup strains, respectively. SNPs were filtered using the BioNumerics strict SNP filtering template. CgMLST and wgSNP spanning tree were created in BioNumerics using categorical differences and the unweighted-pair group method with arithmetic mean.

### Virulence and Resistance Gene Profiles

For virulence identification, the 151 isolates were analyzed using the virulence factor database (VFDB)^2^ on August 15th, 2021. The analysis was performed with a minimum 75% identity and 60% coverage.

For resistance gene identification, the 151 isolates were analyzed using ResFinder 3.0 (Center for Genomic Epidemiology). Genes involved in pathogenicity islands, internalins, adherence, invasion, stress, intracellular growth, immunomodulator, peptidase function, immune evasion and bile resistance were investigated. The analysis was performed with a minimum 90% identity and 60% coverage.

### Nucleotide Sequence Accession Number

These assembled genomes were uploaded to NCBI under the Bioproject ID PRJNA759341, accession SAMN21163725-SAMN21163875.

## Results

### Origins of Isolates

One hundred and fifty-one isolates were isolated from 120 cases between 2014 and 2018 in Beijing, China. The origins of the cases are summarized in Table 1. Sixty-three cases were pregnancy-associated infections, in which all mothers were cured; 32 neonates survived, and 28 fetuses died in the womb or after birth. No data were available for three fetuses. Most of the 57 non-pregnancy-associated patients were older people, or patients with various underlying diseases such as cancer and autoimmune disease. The median age of patients with non-pregnancy-associated infections was 46 years old, 18 patients were > 60 years old and 10 patients were < 6 years old. Thirty-one non-pregnancy-associated patients were males and 26 were females. Among the 57 non-pregnancy-associated patients, 35 patients were cured, 7 patients died and 15 patients were lost during follow-up. Blood (65.8%) was the largest sample source. The sample source for 13 cases was cerebrospinal fluid (10.8%) and for 18 pregnancy-associated cases it was placenta (15.0%). Ten samples were from other sources, including neonate pharyngeal smear and external ear canal, infant cord blood, cervical smear, amniotic fluid, ascites, pleural effusion, cystic fluid, bone marrow and subcutaneous drainage.

**TABLE 1:** Characteristics of 120 isolates isolated from 120 cases in Beijing, China, between 2014 and 2018.

Characteristics	Pregnancy-associated, no (%)	Non-pregnancy-associated, no (%)	Total, no.(%)
Total	63 (52.5)	57 (47.5)	120 (100.0)
**Age**			
Newborns and fetus	12 (19.0)	0	12 (10.0)
≤ 20	0	13 (22.8)	13 (10.8)
21∼40	50 (79.4)	13 (22.8)	63 (52.5)
> 40	1 (1.6)	31 (54.4)	32 (26.7)
**Gender**			
Female	63 (100.0)	26 (45.6)	89 (74.2)
Male	0	31 (54.4)	31 (25.8)
**Specimen type**			
Blood	38 (60.3)	41 (71.9)	79 (65.8)
CSF*	2 (3.2)	11 (19.3)	13 (10.8)
Placenta	18 (28.6)	0	18 (15.0)
Other	5 (7.9)	5 (8.8)	10 (8.3)
**Outcome**			
Survival	32 (50.8)	36 (63.2)	68 (56.7)
Death or fetal loss	28 (44.4)	6 (10.5)	34 (28.3)
Unknown	3 (4.8)	15 (26.3)	18 (15.0)
**Serotype**			
1/2a,3a	21 (33.3)	36 (63.2)	57 (47.5)
1/2b,3b,7	35 (55.6)	14 (24.6)	49 (40.8)
4b,4d,4e	7 (11.1)	7 (12.3)	14 (11.7)
**ST^#^**			
ST8	8 (12.7)	14 (24.6)	22 (18.3)
ST87	13 (20.6)	7 (12.3)	20 (16.7)
ST5	11 (17.5)	1 (1.8)	12 (10.0)
ST619	5 (7.9)	4 (7.0)	9 (7.5)
ST155	0	8 (14.0)	8 (6.67)
ST121	4 (6.3)	4 (7.0)	8 (6.67)
Others	22 (34.9)	19 (33.3)	41 (34.2)
**Pulsotypes**			
GX6A16.BJ0012	10 (15.9)	1 (1.8)	11 (9.2)
GX6A16.BJ0003	7 (11.1)	9 (15.8)	16 (13.3)
GX6A16.BJ0044	4 (6.3)	1 (1.8)	5 (4.2)
GX6A16.BJ0132	4 (6.3)	0	4 (3.3)
GX6A16.BJ0176	0	4 (7.0)	4 (3.3)
GX6A16.BJ0013	2 (3.2)	2 (3.5)	4 (3.3)

*CSF, cerebrospinal fluid; ^#^ST, multilocus sequence typing (MLST) types.

### Distribution of Serotypes, Multilocus Sequence Typing and Pulsed-Field Gel Electrophoresis Types

**FIGURE 1:**
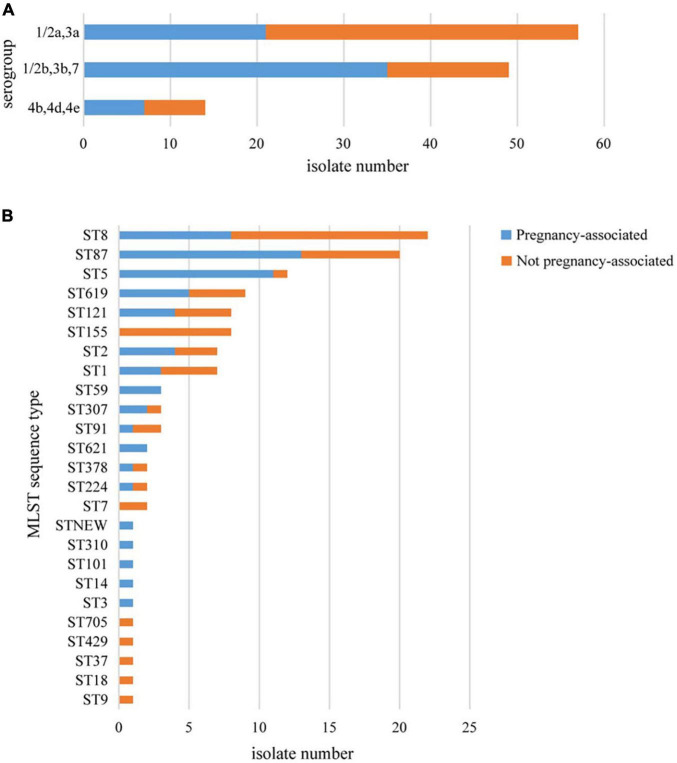
Basic epidemiological characteristics of 120 *L. monocytogenes* isolates isolated from 120 cases in this study. The distribution of isolates is shown according to their molecular serogroup **(A)** and their MLST sequence type **(B)**.

More than one isolate were isolated from different samples at different times (2 cases) or different locations (17 cases) in each of the 19 cases. Among the 19 cases, 11 were mother-infant cases. Isolates from the same cases had the same serogroup, antimicrobial susceptibilities, PFGE types (PTs), and STs. Therefore, only one isolate from each patient was used for these analyses. Almost half of the strains belonged to serogroup 1/2a,3a (*n* = 57, 47.5%), followed by serogroup 1/2b,3b,7 (*n* = 49, 40.8%) and serogroup 4b,4d,4e (*n* = 14, 11.7%). Serogroup distribution differed between pregnancy-associated cases and non-pregnancy-associated cases (Figure 1A). Serogroup 1/2a,3a was more common in non-pregnancy-associated cases, and serogroup 1/2b,3b,7 was more common in pregnancy-associated cases. One hundred and twenty isolates belonged to 25 STs, with one new ST designated (Figure 1B). ST8 (22 isolates, 18.3%), ST87 (20 isolates, 16.7%) and ST5 (12 isolates, 10.0%) were the most frequent STs, followed by ST619 (9 isolates, 7.5%), ST155 (8 isolates, 6.7%), ST121 (8 isolates, 6.7%), ST1 (7 isolates, 5.8%) and ST2 (7 isolates, 5.8%). The other 17 STs contained between one and three isolates. The distribution of STs differed between pregnancy-associated cases and non-pregnancy-associated cases. All ST155 strains were isolated from non-pregnancy-associated patients, whereas 11 of the 12 ST5 isolates were linked to pregnancy-associated infection. One hundred and twenty isolates belonged to 59 PTs, and GX6A16.BJ0003 (16 isolates, 13.3%) was the most frequent PT, followed by GX6A16.BJ0012 (11 isolates, 9.2%), GX6A16.BJ0044 (5 isolates, 4.2%), GX6A16.BJ0132 (4 isolates, 3.3%), GX6A16.BJ0176 (4 isolates, 3.3%) and GX6A16.BJ0013 (4 isolates, 3.3%). The distribution of PTs differed between pregnancy-associated cases and non-pregnancy-associated cases (Table 1). Most GX6A16.BJ0012 isolates (CC87) were from pregnancy-associated cases. All GX6A16.BJ0132 isolates (CC5) were from pregnancy-associated cases, while all GX6A16.BJ0176 isolates (CC87, CC2) were from non-pregnancy-associated cases.

### Cluster Detection Using Core Genome MLST

**FIGURE 2:**
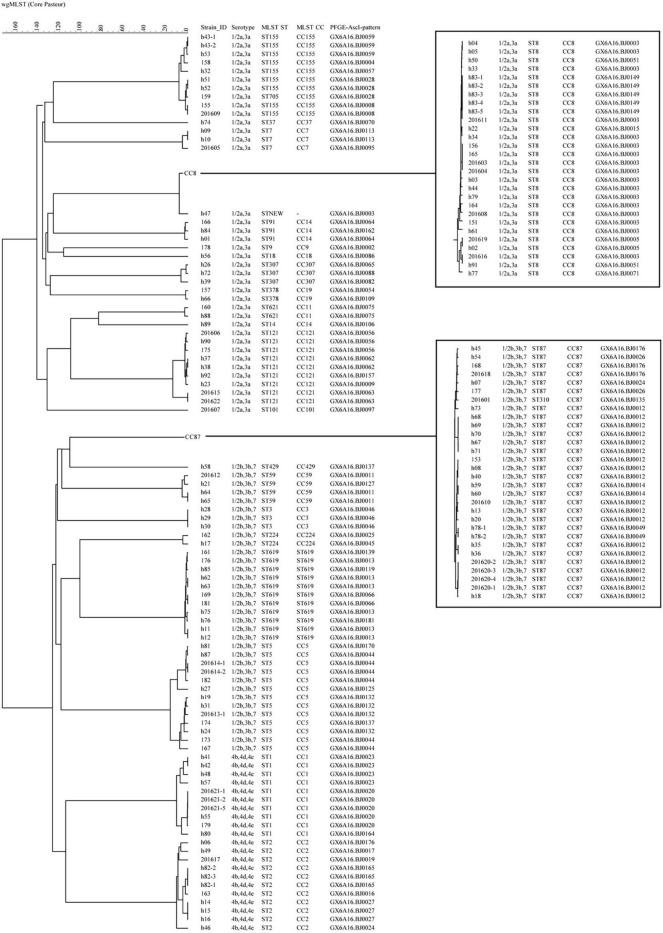
The phylogenomic tree of 151 *L. monocytogenes* isolates based on cgMLST (Core-genome MLST) (*n* = 1,748). The corresponding data, including the name of the isolates (Strain ID), serogroups, MLST type (ST), MLST clonal complexes (CC), PFGE types (PT), were shown alongside the dendrogram to the right.

All 151 isolates were divided into 116 cgMLST types (CTs; Figure 2). Twenty-four CTs contained more than one isolate. Of 17 CTs among these 24 CTs, each CT contained strains isolated from the same patient, with 13 pregnancy-associated cases and 4 non-pregnancy-associated cases, albeit from different sample sources or different sample times. The same CT contained up to five isolates belonging to a single patient. Not all isolates from the same patient had the same CTs. Isolates from the same patient for three cases had a single allele difference in cgMLST. Eighty-six isolates formed 30 complexes with ≤ 7 different alleles between a pair of neighboring isolates (Supplementary Figure 1). The 30 complexes contained between 2 and 13 isolates, between 1 and 8 cases, and between 1 and 4 PTs, but isolates in the same complex had the same STs (Table 2). Isolates in 16 complexes were from two or more cases, indicating possible outbreaks. Among them, seven complexes had no different alleles (C1, C3, C6, C7, C9, C12, C24). In C1, these two strains were isolated from separate patients in the same hospital, but more than 7 months apart. In C6 and C7, the two strains were isolated from different years. WgSNP analysis carried on the 86 isolates yielded similar results to cgMLST (Supplementary Figure 2). Using wgSNP ≤ 12 as a cutoff, eight complexes were detected with more than one case (Table 2).

**TABLE 2:** Relatedness of isolates as determined by cgMLST (≤ 7 different alleles).

Complexes	Possible outbreaks	Isolates	Number of cases	Maximum number of cgMLST alleles	Maximum number of SNPs	ST	Serogroup
C1	O1	201615, 201622	2	0	0	121	1/2a,3a
C2		h37, h38	1	0	0	121	1/2a,3a
C3	O2	h51, h52	2	0	0	155	1/2a,3a
C4	O3	155, 201609	2	2	4	155	1/2a,3a
C5		h43-1, h43-2	1	0	0	155	1/2a,3a
C6	O4	h02, 201619	2	0	0	8	1/2a,3a
C7	O5	h91, 201616	2	0	1	8	1/2a,3a
C8	O6	h05, h04, h50, h03, h33, h22, h34, h83-1, h83-2, h83-3, h83-4, h83-5, 201611	8	7	34	8	1/2a,3a
C9	O7	201603, 201604, 156, 165	3	0	3	8	1/2a,3a
C10		h09, h10	1	1	2	7	1/2a,3a
C11	O8	h08, h40, 153	3	3	6	87	1/2b,3b,7
C12	O9	h59, h60	2	0		87	1/2b,3b,7
C13	O10	h13, 201610	2	7	15	87	1/2b,3b,7
C14		h78-1, h78-2	1	0	0	87	1/2b,3b,7
C15		h35, h36	1	0	0	87	1/2b,3b,7
C16	O11	201620-1, 201620-2, 201620-3, 201620-4, h18	2	5	7	87	1/2b,3b,7
C17		h67, h68, h69, h70, h71	1	1	1	87	1/2b,3b,7
C18		h64, h65	1	0	0	59	1/2b,3b,7
C19		h28, h29, h30	1	0	0	3	1/2b,3b,7
C20	O12	161, 176	2	5	18	619	1/2b,3b,7
C21		h62, h63	1	0		619	1/2b,3b,7
C22	O13	h75, h76	2	7	9	619	1/2b,3b,7
C23		h11, h12	1	0	0	619	1/2b,3b,7
C24	O14	169, 181	2	0	0	619	1/2b,3b,7
C25	O15	h81, h87	2	3	4	5	1/2b,3b,7
C26		201614-1, 201614-2	1	0	0	5	1/2b,3b,7
C27	O16	201621-1, 201621-2, 201621-5, h55, 179	3	4	6	1	4b,4d,4e
C28		h41, h42	1	0		1	4b,4d,4e
C29		h82-1, h82-2, h82-3	1	1	1	2	4b,4d,4e
C30		h14, h15, h16	1	0	0	2	4b,4d,4e

### Typing Resolution of Different Typing Methods

All 151 isolates were grouped into 2 lineages, 3 serogroups, 25STs, 59PTs and 116 CTs. The discriminatory power of cgMLST is apparently superior to the other four types. For example, 62.1% (18) PTs with ≥ 2 isolates (29 PTs) could be further differentiated by cgMLST. Isolates in five PTs with the same CTs were from different patients, and isolates in six PTs with the same CTs were from the same patients. By contrast, only one CT with ≥ 2 isolates could be further discriminated by PFGE, and there was only one pattern difference between these two isolates.

### Comparison With Isolates in the United States

**FIGURE 3:**
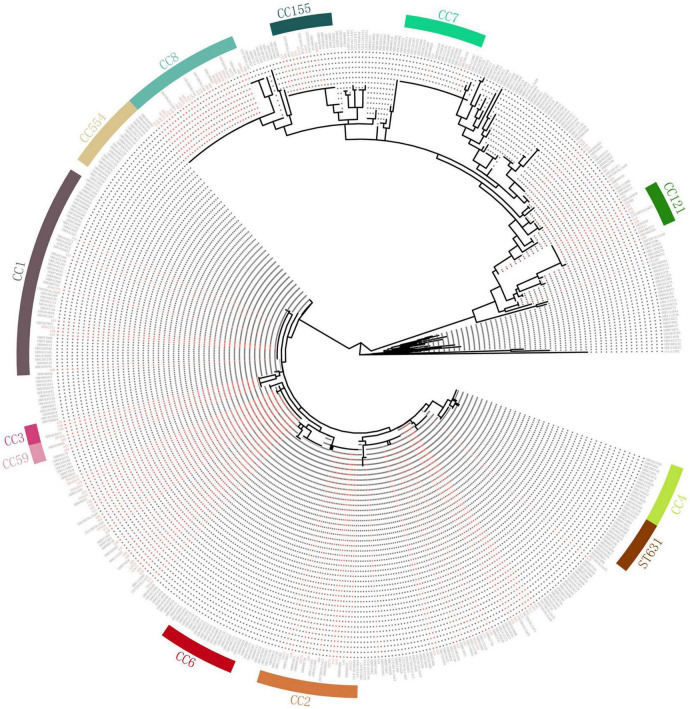
Comparison with isolates in the United States. Clustering of 151 *L. monocytogenes* isolates in our study and 368 human *L. monocytogenes* isolates from the United States based on single-linkage analysis of the cgMLST profiles. Red color represents isolates in our study.

Compared with genomes of 368 human isolates from the United States downloaded from Genbank (Moura et al., 2016), the cgMLST results showed that some of our isolates were dominant in several clusters (Figure 3). Almost all isolates of ST8, ST121, ST619, ST87, and ST155 in our study were clustered with no or few isolates from the United States. Also, there were some Chinese isolates located with the same cluster with isolates from the United States. However, no complexes with ≤ 7 cgMLST alleles were found between isolates of China and the United States.

### *Listeria* Pathogenicity Islands

**FIGURE 4:**
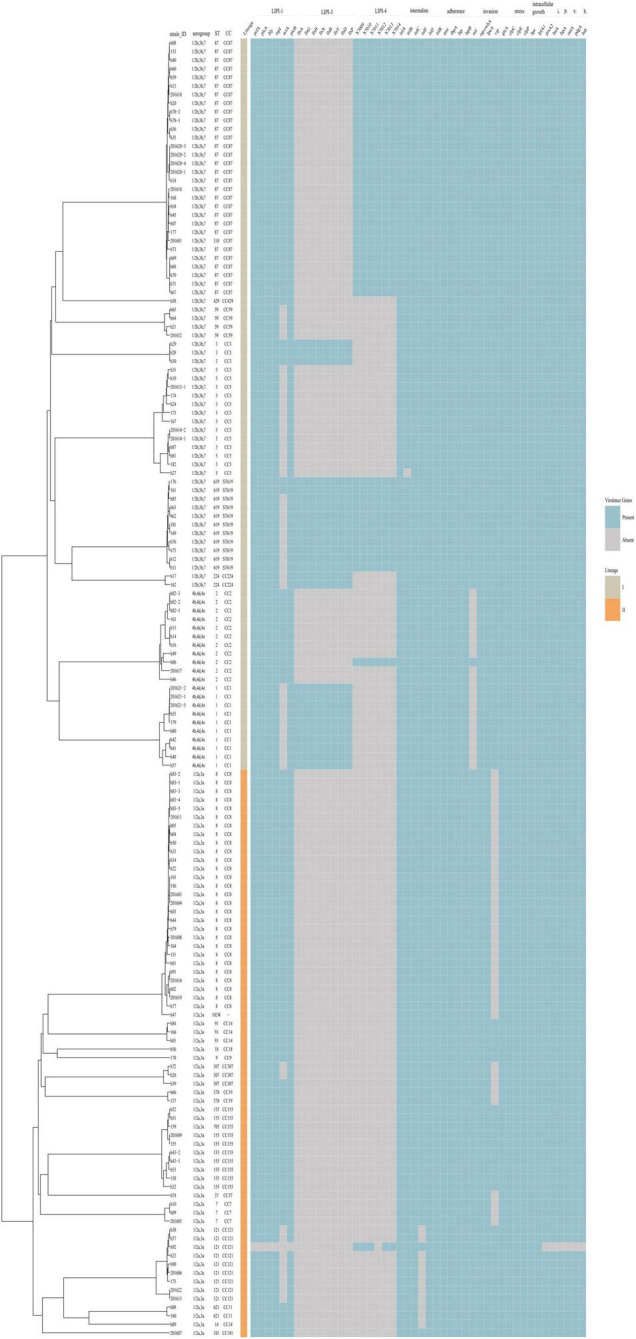
Virulence profiles across the phylogeny of the 151 *L. monocytogenes* isolates. The presence/absence gene matrix represents, from left to right, genes located in the pathogenicity islands LIPI-1 (*prfA, plcA, hly, mpl, actA, plcB*), LIPI-3 (*llsAGHXBYDP*) and LIPI-4 (LM9005581_70009 to LM9005581_70014), genes coding for internalins (*inlABCFGK*) and other genes involved in adherence (*ami, fbpA, lap, lapB*), invasion (*aut, cwhA, lpeA, vip, gtcA*), stress (*clpC, clpE, clpP*), intracellular growth (*hpt, lplA1, prsA2*), immunomodulator (*lntA*), peptidase (*lspA*), immune evasion (*oatA, pdgA*) and bile-resistance (*bsh*).

The virulence and stress resistance gene results revealed important differences between lineage I and lineage II (Figure 4). As expected, the major pathogenicity island LIPI-1 was highly conserved except in one isolate (h92). LIPI-3 was present in some lineage I isolates (16 1/2b,3b,7 isolates and 10 4b,4d,4e isolates) but no lineage II isolates. LIPI-3 was present in all clonal complex (CC) 224, CC3, ST619 and CC1 isolates. Forty-two isolates (41 serogroup1/2b,3b,7 and 1 serogroup 4b,4d,4e) had a complete LIPI-4 recently described and one isolate (h92, serogroup 1/2a,3a) had an incomplete LIPI-4 with no *x7012* gene. Among these isolates, 13 were from 12 non-pregnancy-associated cases, and the other 29 isolates were from 19 pregnancy-associated cases. The presence of LIPI-4 was confirmed in all CC87, all ST619, and one CC2 isolates. Eleven ST619 isolates were confirmed to have both LIPI-3 and LIPI-4. Isolate h92 (1/2a,3a, CC121) had no LIPI-1 but an incomplete LIPI-4 lacking the *x7012* gene.

All isolates encoded full-length *inlA* except one isolate (182). *inlC*, *J* and *K* were present in all isolates, while *InternalinB* was found in all but one isolate. *inlF* was present in almost all isolates except 11 1/2a,3a isolates (8 CC121, 2 CC11, 1 CC14). Genes involved in adherence (*ami*, *fbpA*, *lap*, *lapB*) were present in all isolates. Three of the genes involved in invasion (*cwhA*, *lpeA*, *gtcA*) were present in all isolates, while *aut* was detected in all isolates with the exception of 4b,4d,4e isolates, and *vip* was found in some of the 1/2a,3a isolates.

### Antimicrobial Resistance and Antibiotic Genes

**FIGURE 5:**
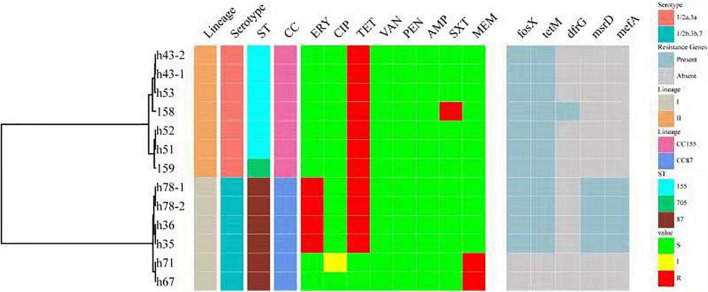
A summary figure showing the lineage, serogroup, ST, CC, and the antimicrobial resistance phenotypes and genotypes of 13 resistant *L. monocytogenes.* ST, MLST type; CC, clonal complex; ERY, erythromycin; CIP, ciprofloxacin; TET, tetracycline; VAN, vancomycin; PEN, penicillin; AMP, ampicillin; SXT, trimethoprim-sulfamethoxazole; MRP, meropenem. Antibiotic resistance genes include *tet*M (encoding resistance to TET), *drf*G (encoding resistance to SXT) and *msr*D and *mef*A (encoding resistance to ERY).

All 151 isolates were found to be susceptible to CIP, VAN, PEN and AMP. Resistance to the other four antibiotics was as follows: TET (11 isolates, 7.28%), ERY (4 isolates, 2.65%), MRP (2 isolates, 1.32%) and SXT (1 isolates, 0.66%). Thirteen isolates (8.6%) were resistant to at least one antibiotic. Four isolates were found to be resistant to two antibiotics. No isolate was defined as multidrug-resistant (MDR). Antibiotic resistance genes were identified in 11 of the 13 isolates (Figure 5). Resistance phenotypes correlated with genotypes, with the exception of MRP resistance, since two isolates were resistant to MRP, but no genes known to encode resistance to MRP were detected. Some resistance genes were identified, including *tet*M (encoding resistance to TET), *drf*G (encoding resistance to SXT), *msr*D and *mef*A (encoding resistance to ERY). As shown in Figure 5, all resistant isolates belonged to serogroup 1/2a,3a and 1/2b,3b,7, CC155 and CC87.

## Discussion

In this study, analysis of 5 years of data (2014−2018) on clinical *L. monocytogenes* isolates in Beijing, China, showed that pregnancy-associated cases and non-pregnancy-associated cases accounted for half each. Listeriosis is an important disease that needs attention and continuous surveillance. Some listeriosis and deaths might go undiagnosed and unreported (Anand et al., 2016; Kylat et al., 2016; Fan et al., 2019). Serogroups 1/2a,3a and 1/2b,3b,7 were the dominant serogroups in our study, consistent with food source strains in China, but different from clinical source strains from other countries (Mammina et al., 2009; Centers for Disease Control and Prevention, 2014; Chen et al., 2015; Maury et al., 2016; Wu et al., 2016; Jennison et al., 2017). In many countries such as the United States, Australia, France and Italy, serotype 4b is the most commonly identified serotype of clinical *L. monocytogenes* (Mammina et al., 2009; Centers for Disease Control and Prevention, 2014; Jennison et al., 2017). The distribution of STs in clinical *L. monocytogenes* strains in Beijing, China differs from those in other countries (Maury et al., 2016; Bergholz et al., 2018; Halbedel et al., 2018). The three most common STs were ST8, ST87 and ST5. While ST8 is distributed globally and ST5 caused several outbreaks in the United States in recent years (Lomonaco et al., 2013; Centers for Disease Control and Prevention, 2015; Jackson et al., 2015), ST87 is seldom linked to human infection in other countries. Approximately 7.5% of STs were ST619 (9 isolates), which has been seldom reported in other countries. The possible reasons for these differences in serogroups and STs are that some clonal groups may not be disseminated widely because of their own characteristics, also there are different types of circulating food, production links, processing techniques and eating habits among regions, resulting in relatively unique clonal groups in different regions. However, some STs prevalent worldwide such as ST1, ST2, ST9, ST121 and ST155 were also identified in this study.

We present a collection of genome-sequenced *L. monocytogenes* isolates from listeriosis patients in Beijing, China, over 5 years. Typing resolution increased from serogroups (3) to MLST (25 STs) to PFGE (59 PTs) to cgMLST (116 CTs), which is consistent with other studies (Kwong et al., 2016; Moura et al., 2016; Halbedel et al., 2018). In our study, most PTs with ≥ 2 isolates could be further differentiated by cgMLST. Only two isolates with the same CTs were further discriminated by PFGE, with one pattern difference. Genomic variation outside the core genome or variations in intergenic region may account for this variability in isolates with identical CTs. In our study, a maximum of one cgMLST locus and two wgSNP loci were found in isolates from the same case isolated in different sample sources or at different sample times that were expected to be highly related. Each of seven CTs included two or three cases, indicating that isolates in the same CTs are likely to be phylogenetically linked. However, following further retrospective investigation, no epidemiological links between the cases in the same CTs could be determined. Previous research (Moura et al., 2016) showed that most isolates sampled during investigations of single outbreaks had seven or fewer allelic mismatches, and isolates with no documented epidemiological links typically differed by more than 10 mismatches. In our study, we used cgMLST ≤ 7 as a cutoff for the classification of closely linked isolates, and 16 complexes were identified. When wgSNP ≤ 12 was used as a cutoff, eight complexes were found. These results imply that strains in the same complex should arise the potential for common source outbreaks. CgMLST is supposed to become a universal tool for cluster detection and international communication during regional or global listeriosis outbreaks because it improves *L. monocytogenes* typing and reduces unnecessary epidemiological investigations (Moura et al., 2016). CgMLST typing results do not require a multiple sequence alignment step, and they are easier to be interpreted by microbiologists, epidemiologists and public health professionals (Moura et al., 2016). Simplified cgMLST data can be readily exchanged between global laboratories (Halbedel et al., 2018). However, linking *L. monocytogenes* isolates to listeriosis outbreaks without epidemiological data is not feasible (Hilliard et al., 2018).

The use of cgMLST analysis helps to determine the population structure, and indicates cross-country and intercontinental transmission of *L. monocytogenes* (Moura et al., 2016). To date, only a few cross-country outbreaks have been recognized (Schmid et al., 2014; Wang et al., 2018), and most listeriosis outbreaks have gone unreported (Leclercq et al., 2014). In our study, we compared isolates with those from the United States. No closely linked isolates were found between the United States and the isolates in our study, suggesting lack of the transmission behavior of *L. monocytogenes*. The results revealed some isolates located in the same cluster with those from the United States, suggesting long-standing widespread dispersion. The results also demonstrated that ST87 and ST169 were seldom linked to human listeriosis in the United States. Consistent with our results, a study comparing ST87 isolates from various regions of the world showed that the core gene sequence-based phylogeny grouped the majority of clinical and food isolates from China into a unified clade (Yin et al., 2020).

As discussed above, WGS can be used to identify the presence of genes or pathogenicity islands associated with hypervirulence or particular modes of pathogenesis (Maury et al., 2016). In our study, LIPI-1 was present in almost all isolates, consistent with other studies (Hilliard et al., 2018). LIPI-3 plays a role in gastrointestinal colonization (Quereda et al., 2016), and is strongly associated with lineage I strains (Hilliard et al., 2018). In our study, LIPI-3 was detected in 26 lineage I strains, including CC224, CC3 and ST619 of serotype 1/2b,3b,7, and CC1 of serotype 4b,4d,4e. As shown in other studies (Hilliard et al., 2018), there was no LIPI-3 in CC2 of 4b,4d,4e strains. LIPI-4 was recently described as a gene cluster involved in neural and placental infection (Maury et al., 2016), and it appears to be strongly associated with CC4 isolates (Maury et al., 2016; Hilliard et al., 2018). In our study, there were no CC4 isolates, and this island appears to be associated with CC87 and ST619 isolates, and neural and placental infection. Among 19 pregnancy-associated patients with isolates possessing LIPI-4, 6 (31.6%) patients had abortion or fetal death, 11 (57.9%) patients were cured or infants survived, and the outcome of two cases was unknown. Among 44 pregnancy-associated patients with isolates without LIPI-4, 22 (50.0%) patients had abortion or fetal death, 21 (47.7.0%) patients were cured or infants survived, and the outcome of two cases was unknown. The outcomes of patients with isolates possessing LIPI-4 were different, suggesting that other factors contributing to virulence remain to be characterized. Clinical ST619 isolates were especially specific to China (Wang et al., 2018; Zhang et al., 2019), and harbored the most virulence genes. ST619 isolates carried many virulence genes, including *llsX* and ptsA that were also found in various food products in China (Wang et al., 2018, 2021; Chen et al., 2019a,b, 2020). However, to date, little information is available on the pathogenicity of ST619 strains, which should be focused on in future studies.

In this study, 13 (8.6%) of the 151 *L. monocytogenes* isolates were resistant to at least one of the tested antibiotics that are commonly used to treat listeriosis in animals and humans in China. Using WGS, antibiotic resistance phenotypes were established. Antibiotic resistance of *L. monocytogenes* is not as serious as that in *Salmonella*, *E. coli*, *Campylobacter* and some other organisms. Previous study showed that the frequency of acquired resistance in clinical isolates is low, such as in France since 1926 (1.27%), in Poland between 1997 and 2013 (0.29%), resistance is more commonly observed in animal and food isolates (Morvan et al., 2010; Wieczorek and Osek, 2017; Kuch et al., 2018). In a study on 2,862 *L. monocytogenes* isolates from food surveillance in China, the resistance rate for tetracycline (8.7%) was the highest, followed by erythromycin (2.2%), trimethoprim/sulfamethoxazole (0.98%) and chloramphenico (0.8%), similar to our current study (Yan et al., 2019). In this previous study, 11 MDR isolates were identified belonging to ST9, and 13 of the other 17 isolates resistant to trimethoprim/sulfamethoxazole belonged to ST155. In our study, six of the 11 antibiotic resistance isolates belonged to ST155. Two isolates were resistant to MRP, but no genes known to encode resistance to MRP were found. More research is therefore needed.

WGS of *L. monocytogenes* isolates from cases of human listeriosis in Beijing, China, between 2014 and 2018 has provided an overview of locally circulating clinical strains of the pathogen. This work identified particular STs responsible for disease in Beijing, China. CgMLST analysis revealed that isolates from the same patient were highly related, and indicated possible outbreaks, although retrospective follow-up failed to prove any clear epidemiological links. WGS also confirmed the presence of pathogenicity genes or islands and resistance genes.

## Data Availability Statement

The datasets presented in this study can be found in online repositories. The names of the repository/repositories and accession number(s) can be found below: NCBI BioProject, PRJNA759341.

## Author Contributions

XZ, QC, and XM conceived and designed the research study. YN and XM performed the sample collection. XZ and YL performed the experiments. XZ, PZ, and YN analyzed the data. XZ and XM wrote the manuscript. All authors read and approved the final version of the article.

## Conflict of Interest

The authors declare that the research was conducted in the absence of any commercial or financial relationships that could be construed as a potential conflict of interest.

## Publisher’s Note

All claims expressed in this article are solely those of the authors and do not necessarily represent those of their affiliated organizations, or those of the publisher, the editors and the reviewers. Any product that may be evaluated in this article, or claim that may be made by its manufacturer, is not guaranteed or endorsed by the publisher.

## Footnotes

1
http://bigsdb.pasteur.fr/listeria/listeria.html

2
http://www.mgc.ac.cn/VFs/main.htm

## Supplementary Material

Supplementary Figure 1 Minimum spanning tree (MST) of 86 *L. monocytogenes* isolates that formed 30 complexes with ≤ 7 different alleles between a pair of neighboring isolates. Each circle represents a MLST type (ST) and the STs of the isolates were colored as shown in figure. The size of the circle is proportional to the number of the isolates. The shadow zones in different color represent different complexes.

Supplementary Figure 2 Comparison cgMLST analysis with wgSNP carried on the 86 isolates. Numbers on the branch represent the alleles distance. A 33 serogroup 1/2a,3a isolates; B 40 serogroup 1/2b,3b,7 isolates; C 13 serogroup 4b,4d,4e isolates.
