# Supplementary figures and images for: Genomic Characterization of Clinical Listeria monocytogenes Isolates in Beijing, China
Source: Front Microbiol. 2021 Dec 10;12:751003. doi: 10.3389/fmicb.2021.751003 (PMC8703193; doi:10.3389/fmicb.2021.751003)

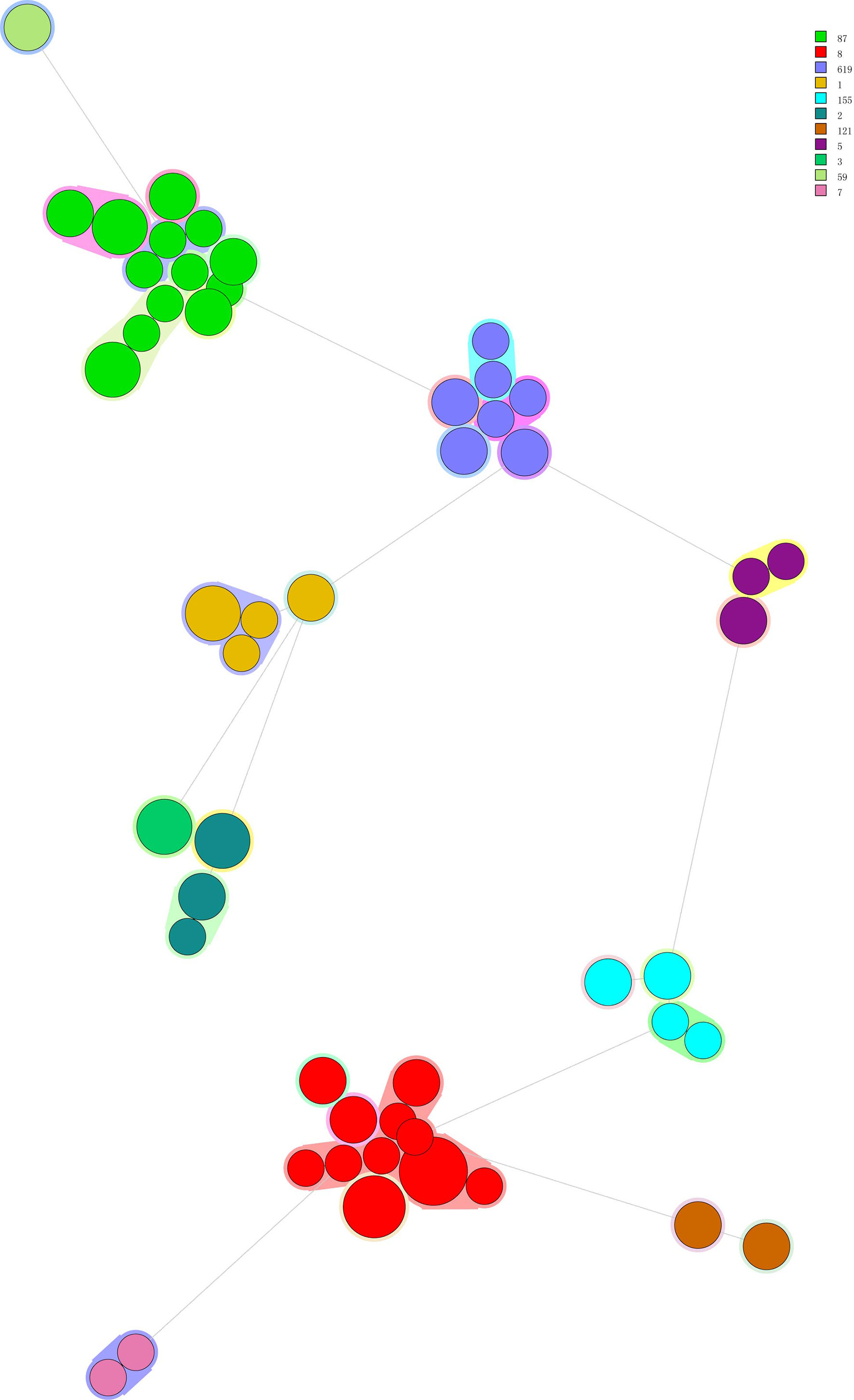

Supplement: Supplementary Figure 1 — Minimum spanning tree (MST) of 86 L. monocytogenes isolates that formed 30 complexes with ≤ 7 different alleles between a pair of neighboring isolates. Each circle represents a MLST type (ST) and the STs of the isolates were colored as shown in figure. The size of the circle is proportional to the number of the isolates. The shadow zones in different color represent different complexes. [file Image_1.JPEG]

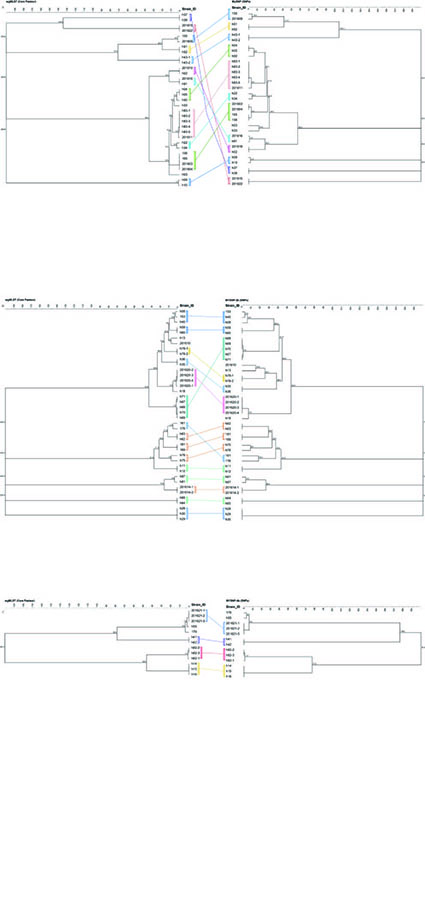

Supplement: Supplementary Figure 2 — Comparison cgMLST analysis with wgSNP carried on the 86 isolates. Numbers on the branch represent the alleles distance. A 33 serogroup 1/2a,3a isolates; B 40 serogroup 1/2b,3b,7 isolates; C 13 serogroup 4b,4d,4e isolates. [file Image_2.JPEG]
